# The Expanding Role of the Infectious Disease Expert in the Context of the MS Centre

**DOI:** 10.3390/jpm12040591

**Published:** 2022-04-07

**Authors:** Matteo Lucchini, Paola Del Giacomo, Valeria De Arcangelis, Viviana Nociti, Assunta Bianco, Chiara De Fino, Giorgia Presicce, Alessandra Cicia, Vincenzo Carlomagno, Massimiliano Mirabella

**Affiliations:** 1 UOC Neurologia, Fondazione Policlinico Universitario Agostino Gemelli IRCCS, 00168 Rome, Italy; v.dearcangelis@gmail.com (V.D.A.); viviana.nociti@policlinicogemelli.it (V.N.); assunta_bianco@yahoo.it (A.B.); chiaradefino@me.com (C.D.F.); cicia.alessandra@gmail.com (A.C.); vincenzo.carlomagno94@gmail.com (V.C.); massimiliano.mirabella@unicatt.it (M.M.); 2 Dipartimento di Neuroscienze, Università Cattolica del Sacro Cuore, CERSM, 00168 Rome, Italy; 3 UOC Malattie Infettive, Fondazione Policlinico Universitario Agostino Gemelli IRCCS, 00168 Rome, Italy; paola.delgiacomo@policlinicogemelli.it; 4 Fondazione Santa Lucia IRCCS, 00179 Rome, Italy; presiccegiorgia@gmail.com

**Keywords:** multiple sclerosis, disease-modifying treatment, infectious diseases, vaccination, chronic infections, progressive multifocal leukoencephalopathy, tuberculosis, hepatitis B, varicella zoster virus, herpes simplex infection

## Abstract

Introduction: The complexity of the MS patient’s management is constantly growing. Consequently, the MS care unit requires a multidisciplinary approach, including an infectious disease specialist to minimise the risk of infectious complications related both to the disease and DMTs. Materials and methods: We retrospectively evaluated the infectious disease consultations performed from 2015 to 2019 in our MS centre. Results: We identified 107 patients with at least one infectious disease consultation out of 1088 patients. We found a progressive increase in the number of consultations from 2015 to 2019. Nearly half of the consultations were requested at the time of starting MS treatment. The most frequent requests were represented by chronic or acute infections. The most prevalent infectious agents were Herpesviridae and Mycobacterium tuberculosis. Antibiotic or antiviral treatment and prophylactic treatment or vaccination represented together the most frequent outcomes of the consultations. Finally, a treatment delay was significantly associated with the advice of a prophylactic treatment or of a vaccination. Conclusion: There is an increasing awareness of the potential infectious complications of MS and of exposure to DMTs. The interaction between the MS neurologist and infectious disease specialist is fundamental to minimise the infectious risk related to the disease and to the DMTs, with a progressive shift from complication management to a broader prevention workup at the time of MS diagnosis, including both vaccination and prophylactic treatments.

## 1. Introduction

Multiple sclerosis (MS) is a chronic demyelinating disease of the central nervous system (CNS) affecting more than two million people worldwide [1].

MS pathogenesis is not fully understood and is thought to be multifactorial, involving a complex interplay between the immune system and different environmental factors [2,3]. Epidemiological studies suggest a potential role for many common infections in the MS aetiology, mainly focusing on EBV infection [4,5,6]. Moreover, some studies have shown that infections can be a trigger for MS relapses and for neurological worsening and progression [7,8].

Overall, MS patients have an increased risk of infections compared with the general population [9,10]. Injectable disease-modifying therapies (DMTs), such as interferon beta and glatiramer acetate, do not seem to significantly increase this risk [11]. In contrast, the new-generation DMTs, including oral immunosuppressive agents and monoclonal antibodies, have been associated with an increased risk of infections compared with placebo or interferon beta and glatiramer acetate, both in randomised clinical trials and in post-marketing surveillance [12].

Each DMT is associated with an increased risk of specific infections. Natalizumab increases the risk for life-threatening progressive multifocal leukoencephalopathy (PML). To a significantly lesser extent, PML has also been associated with exposure to other DMTs [13]. Herpes simplex virus (HSV) and varicella zoster virus (VZV) infections and reactivation have been observed in randomised clinical trials (RCTs) with fingolimod, alemtuzumab, cladribine and ocrelizumab [14]. HBV reactivation can be devastating for patients treated with alemtuzumab or with anti-CD20 monoclonal antibodies [15]. A specific diet and a prophylactic antibiotic treatment are recommended before the initiation of alemtuzumab in order to reduce the risk of infection with Listeria monocytogenes [16]. The risk of infection is also increased by specific patient characteristics, such as older ages and higher disability [14]. These immunosuppressive and immunomodulating agents increase the susceptibility to infections, reactivating latent pathogens, worsening asymptomatic chronic infections, and contracting de novo infections, demanding a meticulous safety monitoring.

Considering the complexity of the treatments available and the related infectious risks, the approach to MS patients’ management requires a multidisciplinary team. In recent years, there has been a greater awareness towards an adequate MS care unit, recognising the role of different specialists in MS to ensure a better quality of life through a personalised clinical framework [17].

In this perspective, our study aims to illustrate the evolving role of the infectious disease specialist in the context of the MS care unit to minimise the infection risk related to both MS and DMTs.

## 2. Materials and Methods

### 2.1. Patient Selection

This is a retrospective monocentric study to evaluate the incidence and the outcome of infectious disease counselling in a monocentric cohort of MS patients. We included patients with MS diagnosis followed at the Multiple Sclerosis Unit of Fondazione Policlinico Universitario Agostino Gemelli IRCCS from January 2015 to December 2019. Inclusion criteria included the following: MS diagnosis following the most recent McDonald criteria [18]; at least two neurological evaluations; and at least one infectious disease counselling session within the study period.

For each patient, we collected the following clinical and paraclinical parameters: age, sex, disease duration, EDSS, disease course, and the number and the year of infectious disease counselling.

### 2.2. Infectious Disease Counselling Description

For each infectious disease evaluation, we collected the following data: the timing of the request; the clinical motivation; the outcome of the counselling; and the eventual impact on MS treatment.

Regarding the timing of the evaluation, we distinguished three different moments: the diagnostic workup, treatment initiation or switching, and evaluation during treatment exposure.

We divided the counselling requests into the following categories: isolated serological findings to be interpreted; fungal or parasitic infection; bacterial infection; viral infection; and vaccination.

The outcomes of the counselling were classified as non-specific (interpretation of a result without the need for further interventions); antibiotic/antiviral treatment; vaccination/prophylaxis (the last intended for latent or chronic inactive infections); and treatment authorisation without further analysis.

Finally, the impact on MS treatment was divided into no impact, delay in treatment initiation, and suspension/changing of treatment.

### 2.3. Statistical Analysis

Continuous variables were described as the mean ± standard deviation. Dichotomic or categorical variables were expressed as frequencies. The eventual relationship between categorical variables was explored with the chi-squared test. Eventual associations between specific items of the infectious disease consultation were evaluated through the chi-squared adjusted residual analysis with Bonferroni correction. All two-tailed *p*-values < 0.05 were considered significant. Data were analysed using the Statistical Package for Social Sciences, version 16.0 (IBM SPSS, Inc., Chicago, IL, USA).

## 3. Results

### 3.1. Patients’ Characteristics

We identified 107 patients with at least one infectious disease counselling session out of the 1088 patients who were followed in our MS centre within the study period. Patients’ clinical features are described in Table 1. Briefly, the mean age was 41 years with a female predominance and a widely variable disease duration. Relapsing MS represented the most frequent disease course with a wide range of EDSS scores (from 0 to 8.0). We included 155 unique infectious disease consultations in our analysis. Nearly 35% of the patients underwent more than one infectious disease consultation during the study period. Regarding the year of counselling, we found a progressive increase in the number of consultations from 2015 to 2019.

### 3.2. Infectious Disease Counselling

Nearly half of the consultations were requested at the time of starting MS treatment, while one-third were requested as follow-up evaluations during treatment exposure and only a minority as part of the diagnostic workup (see Table 2).

The most frequent requests were represented by chronic or acute infections (56.8%). Bacterial agents represented the most frequent cause of infection (*n* = 44, 28.4%), followed by viral infection (*n* = 37, 23.9%). The most prevalent infectious agents were Herpesviridae (more than half represented by HSV-1 infection) and Mycobacterium tuberculosis (included in the bacterial infection) with 20 cases each. We found only a few cases of fungal infections, mainly Candida urinary tract infections, and one case of intestinal Giardiasis.

However, nearly 25.2% of the evaluations were requested following isolated serological alterations without evident clinical impact (see Table 3).

Antibiotic or antiviral treatment and prophylactic treatment or vaccination represented together the most frequent outcomes of the consultations (58.1%). In the other cases, the clinical/serological findings were considered non-specific and the treatment was authorised without further examinations (see Table 4). Half of the consultations resulted in a delay in treatment initiation while 41.3% had no impact on the timing of MS treatment initiation (Table 5).

**Figure 1:**
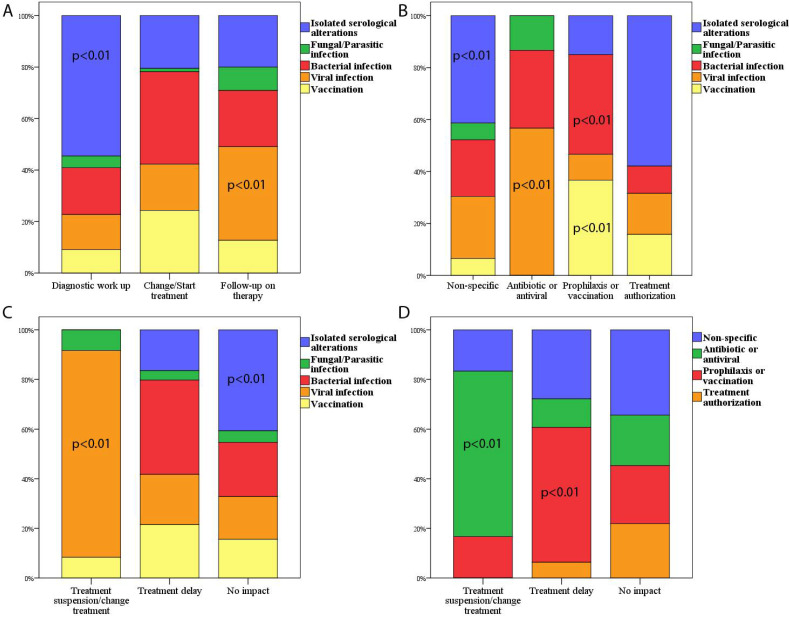
(**A**) A bar chart showing the relative percentage of the type of infectious disease counselling requests divided by the timing of the consultation. (**B**) A bar chart showing the relative percentage of the type of infectious disease counselling requests divided by the specialist’s advice. (**C**) A bar chart showing the relative percentage of the type of infectious disease counselling requests divided by the impact on MS treatment. (**D**) A bar chart showing the relative percentage of the specialist’s advice divided by the impact on MS treatment. In (**A**–**D**) the *p* values below 0.05 of the chi-squared adjusted residual analysis are reported.

In the diagnostic work-up period, the most frequent motivation of infectious disease evaluation was the presence of isolated serological alterations while infections, particularly the viral ones, represented the more prevalent cause of evaluation in patients exposed to DMTs (*p* < 0.01; Figure 1A).

Regarding the relationship between the motivation of the request and the outcome of the consultation, we found that the presence of asymptomatic serological alterations was significantly associated with an interpretation as non-specific or with treatment authorisation without further investigations (*p* < 0.01). Furthermore, a suspected infection (especially for viral infection) resulted in antibiotic/antiviral treatment in most cases (*p* < 0.01), while in the cases of evaluation for eventual vaccination or bacterial latent infections (in most cases latent tuberculosis, *n* = 20), a prophylactic treatment or vaccination advice was given (Figure 1B).

We found a significant association between treatment suspension and viral infection (*p* < 0.01), as viral infections represented the most frequent cause of treatment suspension or switch (10 out of 12). Moreover, isolated serological alterations had no impact on MS treatment in most cases (Figure 1C).

Finally, a treatment delay was significantly associated (*p* < 0.01) with the advice of a prophylactic treatment or of a vaccination, while treatment suspension or change followed the advice of an antibiotic or antiviral treatment (Figure 1D).

## 4. Discussion

In our monocentric study, we retrospectively evaluated 107 patients with MS in a five-year span undergoing infectious disease evaluation to understand the timing, motivation, outcome and impact of this counselling on MS management. The role of an infectious specialist in the MS care unit can be crucial in several situations, such as screening for infection in naive patients, the safety monitoring of patients exposed to DMTs, and the management of respiratory (RTIs) and urinary tract (UTIs) infections that are more prevalent in elderly and more disabled patients [19,20].

In our study, we observed that the number of infectious counselling requests progressively increased from 2015 to 2019. In this timeframe, different highly effective DMTs were approved, including alemtuzumab, ocrelizumab and cladribine, requiring specific baseline infectious evaluations and thus resulting in an increased awareness of the potential infectious risks [12].

Nevertheless, most patients (50.3%) were evaluated by the infectious specialist at the time of starting or changing the treatment with DMTs. Most of these evaluations were driven by the request of a specific vaccination, mainly hepatitis B virus (HBV) and varicella zoster virus (VZV), or of the management of latent/chronic infections (mainly tuberculosis and to a lesser extent chronic inactive HBV) [21,22]. The resulting infectious disease consultation outcomes were represented by the need for specific vaccinations or for prophylactic antibiotic or antiviral therapy before DMT started. This finding represents an important shift in MS care from infectious complication management to a wider prevention work-up at the time of MS diagnosis [23]. Considering the ever-expanding scenario of the available DMTs carrying specific infectious risks, it is fundamental to perform an extensive study of potential infection risk as soon as possible to minimise the risk of life-long exposure to different DMTs [21].

At the time of diagnostic evaluation, data should be collected on physiological and epidemiological factors, occupational history, past medical history of previous infections and vaccinations, and family history, in order to produce a kind of “infectious identity card” [24]. Moreover, these evaluations, and the consequent interventions, should be performed before starting with the therapy since several DMTs could reduce the efficacy and the safety of vaccinations or interfere with other prophylactic strategies [25].

A recent paper from Riva et al. evaluated the relationship between MS and vaccination. In this consensus work, the authors concluded that, despite the need for more research in this specific field, MS patients should be vaccinated before starting DMT, and a specific infectious and immunisation history at diagnosis should be collected [21].

However, vaccination and prophylactic treatments are associated with a treatment delay with potential clinical implications [26].

Some patients (14.2%) required an infectious disease consultation at the time of diagnosis. The presence of isolated serological alterations represents the most important motivation of the counselling request for those patients. These serological alterations were mainly represented by the presence of EBV genome both in serum or in the CSF, the transient finding of IgM specific for HSV and Borrelia burgdorferi, without any specific epidemiological history and or symptoms [27,28]. Despite most of these non-specific serological alterations not affecting the beginning, change or suspension of the therapy, some caused a treatment delay to be properly interpreted.

In our study, we had only one case with an infectious disease consultation required for a progressive multifocal leukoencephalopathy (PML) suspicion, subsequently not confirmed. Strict clinical vigilance to minimise the risk of PML, especially for natalizumab-treated patients, was performed by the neurologist following a stratification risk protocol [29]. The specific awareness and expertise of the MS neurologists in the field should explain this finding. Moreover, in these specific situations, it could be the fundamental role of the neuroradiologist to identify early magnetic resonance (MRI) changes before symptom onset [30].

Furthermore, we required an infectious disease consultation for a human papillomavirus (HPV) infection in one case, since in our setting HPV-related disease is mainly managed together with the gynaecologists. A periodic screening for HPV-related disease is mandatory in MS patients, particularly for those exposed to sphingosine-1-phosphate modulators [31].

Few patients (*n* = 12) in our study suspended DMT after the infectious disease consultation. In most cases, a viral infection (HSV or VZV reactivation) represented an infectious complication leading to treatment discontinuation, as expected for the DMT risk profiles [32].

### Study Limits

We decided to not include 2020 data since the COVID-19 pandemic forcibly increased the number of requests for infectious disease counselling.

The estimation of infection prevalence in MS patients was not an objective of our study since most of the RTIs and UTIs are directly managed by the general practitioner, not requiring a specialist evaluation in the MS centre.

## 5. Conclusions

The new concept of the MS care unit stressed the importance of a multidisciplinary approach in which specific competences facilitate correct treatment and increase the safety for patients. With our study, we underline the increasing importance of the infectious disease specialist as another competence to add to the MS care unit supporting the neurologist in the minimisation of the infectious risk related to DMTs, and in the timely management of eventual complications.

## Figures and Tables

**Table 1:** Patients’ demographics.

Patients	*n* = 107
Female sex, *n* (%)	78 (72.9)
Age, years, mean (SD)	41.4 (14.4)
Disease duration, years, mean (SD)	8.5 (9.4)
EDSS, median [range]	1.5 [0–8.0]
Disease course, *n* (%)	
RMS	78 (72.9%)
SPMS	19 (17.8%)
PPMS	10 (9.3%)
Number of counselling, *n* (%)	
1	69 (64.5%)
2	30 (28.0%)
3	6 (5.6%)
4	2 (1.9%)
Year of counselling, *n* (%)	
2015	5 (4.7%)
2016	8 (7.5%)
2017	22 (20.6%)
2018	26 (24.3%)
2019	46 (43.0%)

EDSS: Expanded Disability Status Scale; RMS: Relapsing Multiple Sclerosis; SPMS: Secondary Progressive Multiple Sclerosis; PPMS: Primary Progressive Multiple Sclerosis; SD: standard deviation.

**Table 2:** Timing of counselling request.

Counselling Timing	*n* = 155
Diagnostic workup	22 (14.2%)
Start or change treatment	78 (50.3%)
Follow-up on treatment	55 (35.5%)

All values are reported as a number (percentage).

**Table 3:** Infectious disease counselling request.

Counselling Motivation	*n* = 155
Isolated serological findings to be interpreted	39 (25.2%)
Fungal or parasitic infection	7 (4.5%)
Bacterial infection	44 (28.4%)
Viral infection	37 (23.9%)
Vaccination	28 (18.1%)

All values are reported as a number (percentage).

**Table 4:** Counselling outcome.

Counselling Timing	*n* = 155
Non-specific	46 (29.7%)
Antibiotic or antiviral treatment	30 (19.4%)
Prophylaxis or vaccination	60 (38.7%)
Treatment authorisation	19 (12.3%)

All values are reported as a number (percentage).

**Table 5:** Counselling impact on MS-specific treatment.

Impact on MS Treatment	*n* = 155
Treatment changed or suspended	12 (7.7%)
Treatment delay	79 (51.0%)
No impact	64 (41.3%)

All values are reported as a number (percentage).

## Data Availability

All data are available to researchers upon request for the purpose of reproducing the results or replicating the procedure by directly contacting the corresponding author.
